# Supplementary material for: LILRB1-HLA-G axis defines a checkpoint driving natural killer cell exhaustion in tuberculosis
Source: EMBO Mol Med. 2024 Jul 19;16(8):1. doi: 10.1038/s44321-024-00106-1 (PMC11319715; doi:10.1038/s44321-024-00106-1)
Supplement: Supplementary file 1 — Appendix [file 44321_2024_106_MOESM1_ESM.pdf]

Appendix

LILRB1-HLA-G axis defines a checkpoint driving natural killer cell exhaustion in tuberculosis

Table of contents：

Appendix Figure S1 2

Appendix Figure S2 4

Appendix Figure S3 6

Appendix Figure S4 8

Appendix Figure S5 10

Appendix Figure S6 12

Appendix Figure S7 14

Appendix Figure S8 16

Appendix Figure S9 18

Appendix Table S1 20

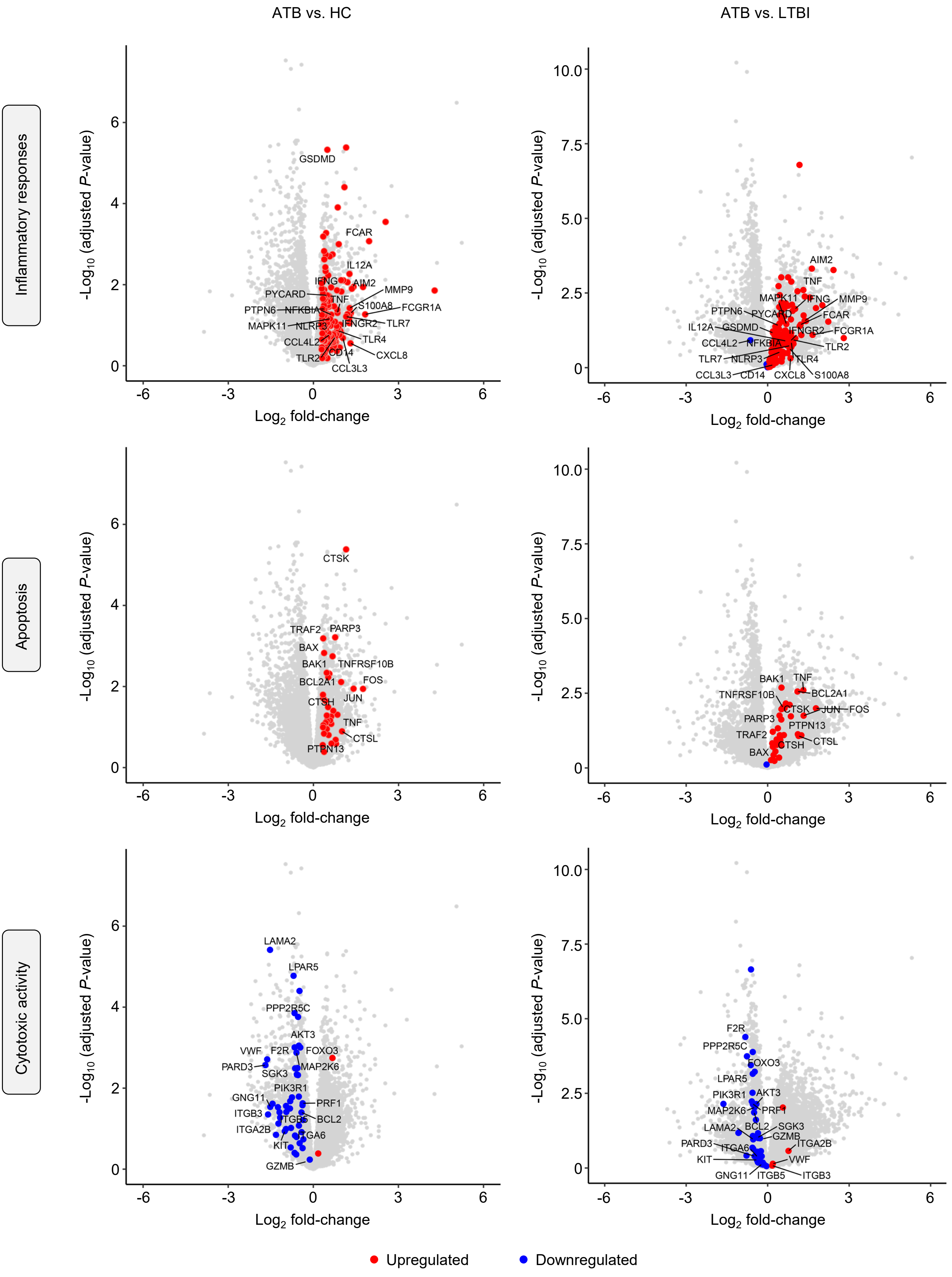

ATB vs. HC
ATB vs. LTBI
Inflammatory responses
Apoptosis
Cytotoxic activity
-Log10 (adjusted P-value)
Log2 fold-change
GSDMD
FCAR
IL12A
IFNG
AIM2
MMP9
PYCARD
TNF
S100A8
FCGR1A
PTPN6
NFKBIA
MAPK11
NLRP3
IFNGR2
TLR7
TLR4
CCL4L2
CD14
CXCL8
TLR2
CCL3L3
IL12A
CTSK
TRAF2
PARP3
BAX
BAK1
TNFRSF10B
BCL2A1
FOS
CTSH
JUN
CTSL
PTPN13
LAMA2
LPAR5
PPP2R5C
AKT3
VWF
F2R
FOXO3
PARD3
MAP2K6
SGK3
PIK3R1
GNG11
PRF1
ITGB3
ITGB5
BCL2
ITGA2B
ITGA6
KIT
GZMB
Upregulated
Downregulated

Appendix Figure S1. Volcano maps depicting differentially expressed genes (DEGs) between HC, LTBI, and ATB groups. Genes involved in inflammatory responses, apoptosis, or cytotoxic activity based on Kyoto encyclopedia of genes and genomes (KEGG) enrichment were indicated in red (upregulated) or blue (downregulated).

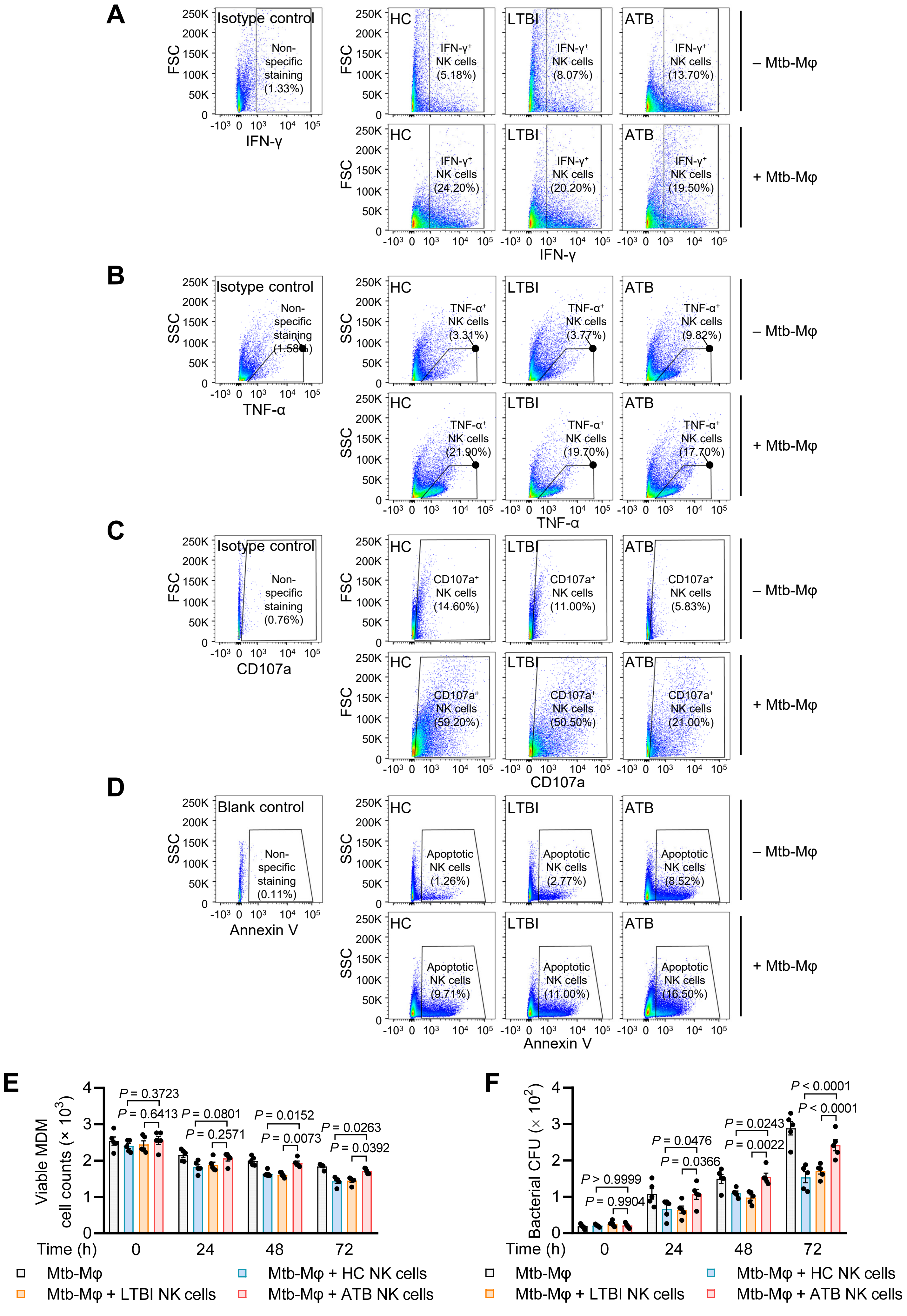
A
B
C
D
E
F
Isotype control
Non-specific staining (1.33%)
IFN-γ
FSC
HC
LTBI
ATB
IFN-γ⁺ NK cells (5.18%)
IFN-γ⁺ NK cells (8.07%)
IFN-γ⁺ NK cells (13.70%)
– Mtb-Mφ
IFN-γ⁺ NK cells (24.20%)
IFN-γ⁺ NK cells (20.20%)
IFN-γ⁺ NK cells (19.50%)
+ Mtb-Mφ
Non-specific staining (1.58%)
TNF-α
SSC
TNF-α⁺ NK cells (3.31%)
TNF-α⁺ NK cells (3.77%)
TNF-α⁺ NK cells (9.82%)
TNF-α⁺ NK cells (21.90%)
TNF-α⁺ NK cells (19.70%)
TNF-α⁺ NK cells (17.70%)
Non-specific staining (0.76%)
CD107a
CD107a⁺ NK cells (14.60%)
CD107a⁺ NK cells (11.00%)
CD107a⁺ NK cells (5.83%)
CD107a⁺ NK cells (59.20%)
CD107a⁺ NK cells (50.50%)
CD107a⁺ NK cells (21.00%)
Blank control
Non-specific staining (0.11%)
Annexin V
Apoptotic NK cells (1.26%)
Apoptotic NK cells (2.77%)
Apoptotic NK cells (8.52%)
Apoptotic NK cells (9.71%)
Apoptotic NK cells (11.00%)
Apoptotic NK cells (16.50%)
250K 200K 150K 100K 50K 0
-10³ 0 10³ 10⁴ 10⁵
Viable MDM cell counts (× 10³)
Time (h)
0 24 48 72
P = 0.3723
P = 0.6413
P = 0.0801
P = 0.2571
P = 0.0152
P = 0.0073
P = 0.0263
P = 0.0392
Bacterial CFU (× 10²)
P > 0.9999
P = 0.9904
P = 0.0476
P = 0.0366
P = 0.0243
P = 0.0022
P < 0.0001
P < 0.0001
Mtb-Mφ
Mtb-Mφ + HC NK cells
Mtb-Mφ + LTBI NK cells
Mtb-Mφ + ATB NK cells

Appendix Figure S2. NK cells from ATB patients show attenuated ability against Mtb infection. (**A**–**D**) Representative results of fluorescence-activated cell sorting (FACS)-based analysis for percentages of IFN-γ^+ cells (**A**), TNF-α^+ cells (**B**), CD107a$^+$ cells (**C**) and apoptotic cells stained with annexin V (**D**) in total NK cells from peripheral blood of individuals in HC, LTBI, or ATB groups. NK cells from individuals in HC, LTBI, or ATB groups were co-cultured with (+) or without (–) Mtb-infected MDMs (Mtb-Mφ) for 24 hours. (**E**) Viable MDM cell counts. (**F**) Mtb survival in MDM-NK cell co-cultures as determined by colony forming unit (CFU) counting. For (**E**, **F**), MDMs infected with Mtb were co-cultured with NK cells from individuals in HC, LTBI, or ATB groups for 0–3 days. Data are mean ± SEM (n = 5 donors per group) in (**E**, **F**). Statistical significance was determined using two-way ANOVA with Tukey's post-hoc test. Results are representative of three independent experiments.

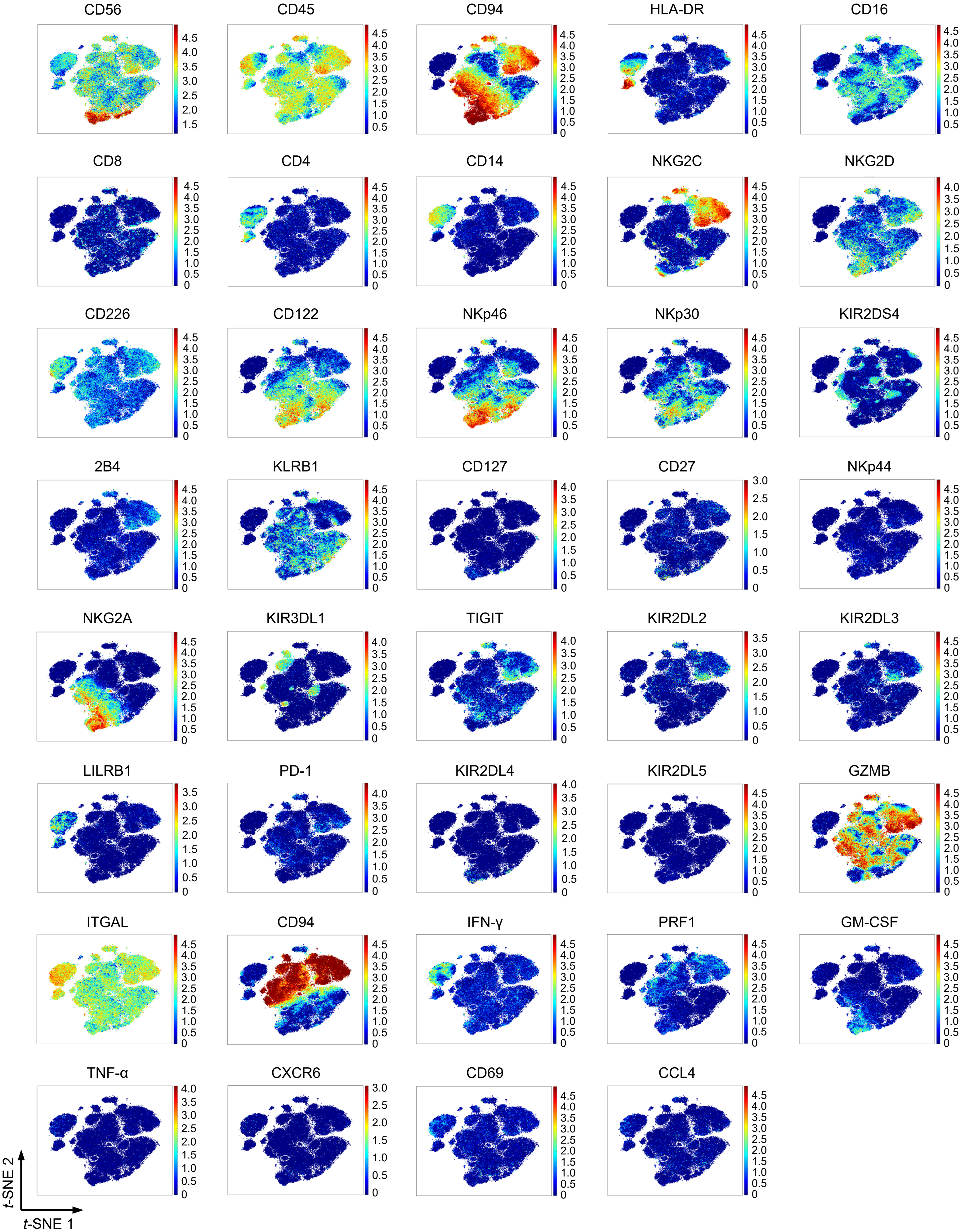

CD56
CD45
CD94
HLA-DR
CD16
CD8
CD4
CD14
NKG2C
NKG2D
CD226
CD122
NKp46
NKp30
KIR2DS4
2B4
KLRB1
CD127
CD27
NKp44
NKG2A
KIR3DL1
TIGIT
KIR2DL2
KIR2DL3
LILRB1
PD-1
KIR2DL4
KIR2DL5
GZMB
ITGAL
CD94
IFN-γ
PRF1
GM-CSF
TNF-α
CXCR6
CD69
CCL4
t-SNE 2
t-SNE 1

Appendix Figure S3. Expression of LILRB1 within different immune cell subsets in HC, LTBI and ATB groups. *t*-SNE maps based on CyTOF single-cell data of NK cells from HC, LTBI, and ATB groups (n = 5 individuals per group) were generated by plotting each event according to its *t*-SNE dimensions in a dot plot, on which the intensities of indicated markers were overlaid to show their expression on different cell subsets (see also Figure 2A).

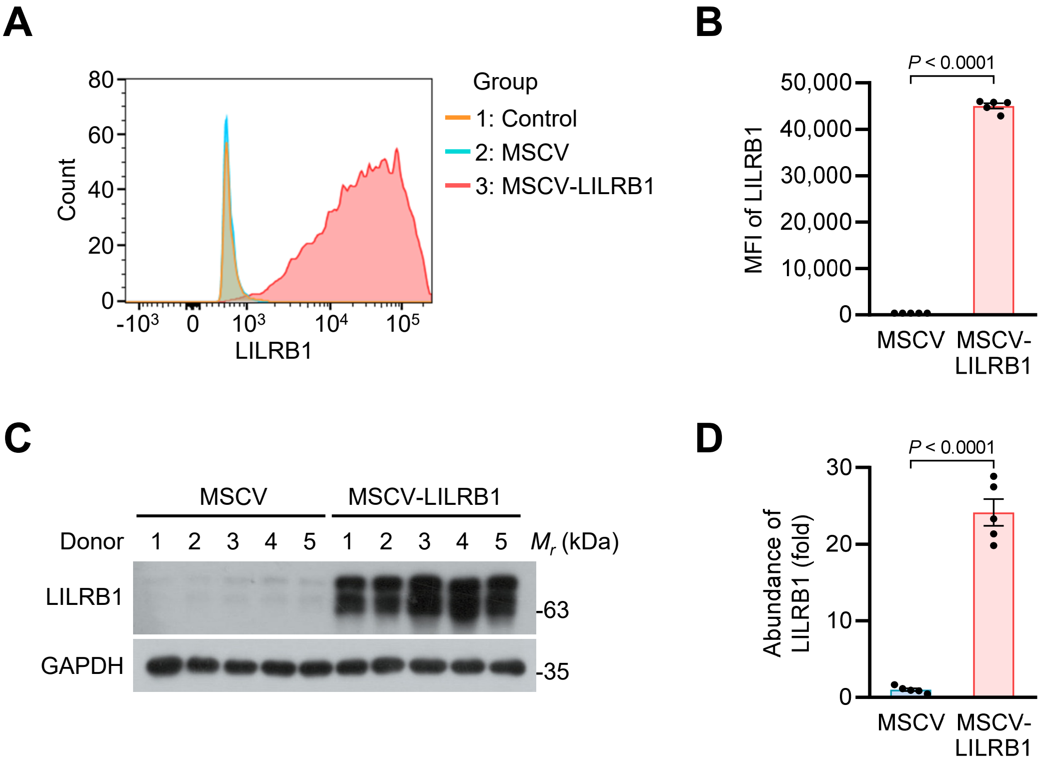
A
B
C
D
Group
1: Control
2: MSCV
3: MSCV-LILRB1
Count
LILRB1
MFI of LILRB1
P < 0.0001
MSCV
MSCV-LILRB1
MSCV
MSCV-LILRB1
Donor 1 2 3 4 5 1 2 3 4 5
Mr (kDa)
LILRB1
-63
GAPDH
-35
Abundance of LILRB1 (fold)
P < 0.0001

Appendix Figure S4. Overexpression of LILRB1 in NK cells. (**A** and **B**) Representative histograms (**A**) and quantitation (**B**) for expression of LILRB1 in HC donor-derived NK cells overexpressing control vector (MSCV) or MSCV-LILRB1. (**C** and **D**) Immunoblotting (**C**) and quantitation (**D**) for expression of LILRB1 in HC donor-derived NK cells overexpressing MSCV or MSCV-LILRB1. Data are mean ± SEM (n = 5 donors per group) in (**B**, **D**). Statistical significance was determined using two-tailed t-test. Results are representative of three independent experiments.

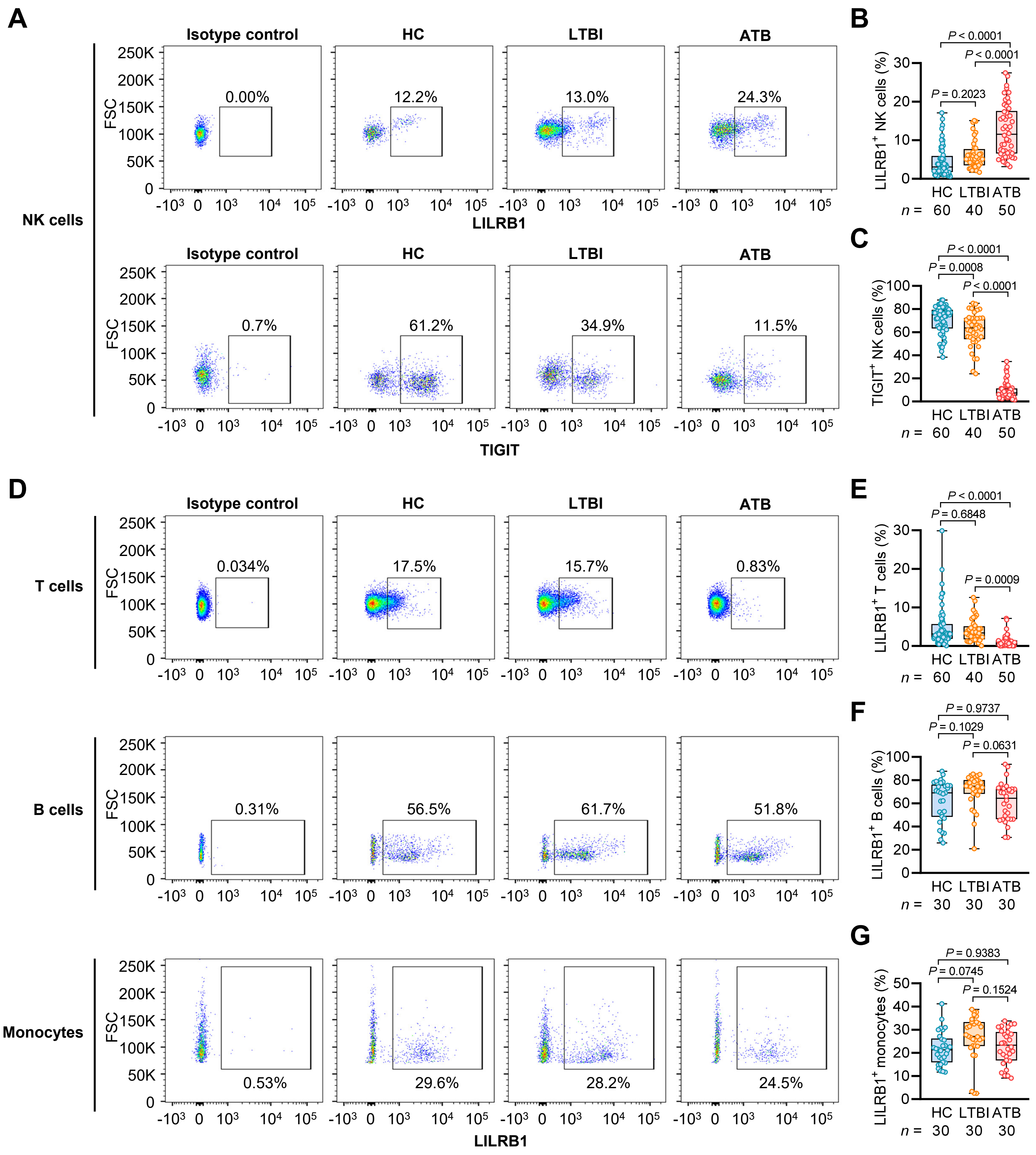
A
Isotype control
HC
LTBI
ATB
NK cells
FSC
LILRB1
0.00%
12.2%
13.0%
24.3%
TIGIT
0.7%
61.2%
34.9%
11.5%
B
LILRB1+ NK cells (%)
P < 0.0001
P < 0.0001
P = 0.2023
HC LTBI ATB
n = 60 40 50
C
TIGIT+ NK cells (%)
P < 0.0001
P = 0.0008
P < 0.0001
HC LTBI ATB
n = 60 40 50
D
T cells
0.034%
17.5%
15.7%
0.83%
B cells
0.31%
56.5%
61.7%
51.8%
Monocytes
0.53%
29.6%
28.2%
24.5%
E
LILRB1+ T cells (%)
P < 0.0001
P = 0.6848
P = 0.0009
HC LTBI ATB
n = 60 40 50
F
LILRB1+ B cells (%)
P = 0.9737
P = 0.1029
P = 0.0631
HC LTBI ATB
n = 30 30 30
G
LILRB1+ monocytes (%)
P = 0.9383
P = 0.0745
P = 0.1524
HC LTBI ATB
n = 30 30 30

Appendix Figure S5. Expression of LILRB1 within different immune cell subsets in HC, LTBI, and ATB groups. (**A**) Representative results of FACS-based analysis for percentages of LILRB1^{+} (upper) or TIGIT^{+} (lower) cells within total NK cells from the peripheral blood of individuals in HC, LTBI, or ATB groups. (**B** and **C**) Percentages of LILRB1^{+} (**B**) or TIGIT^{+} (**C**) cells within total NK cells in the peripheral blood of individuals (*n*) from HC, LTBI, or ATB groups. (**D**) Representative results of FACS-based analysis for percentages of LILRB1^{+} cells within T cells (upper), B cells (middle), or monocytes (lower) from the peripheral blood of individuals in HC, LTBI, or ATB groups. (**E**–**G**) Percentages of LILRB1^{+} cells within total T cells (**E**), B cells (**F**), or monocytes (**G**) in the peripheral blood of individuals (*n*) from HC, LTBI, or ATB groups. Box-whisker plot indicates the interquartile range (box), the median value (line within the box), and the maximum and minimum value (whiskers) in (**B**, **C**, **E**–**G**). Statistical significance was determined using one-way ANOVA with Tukey's post-hoc test.

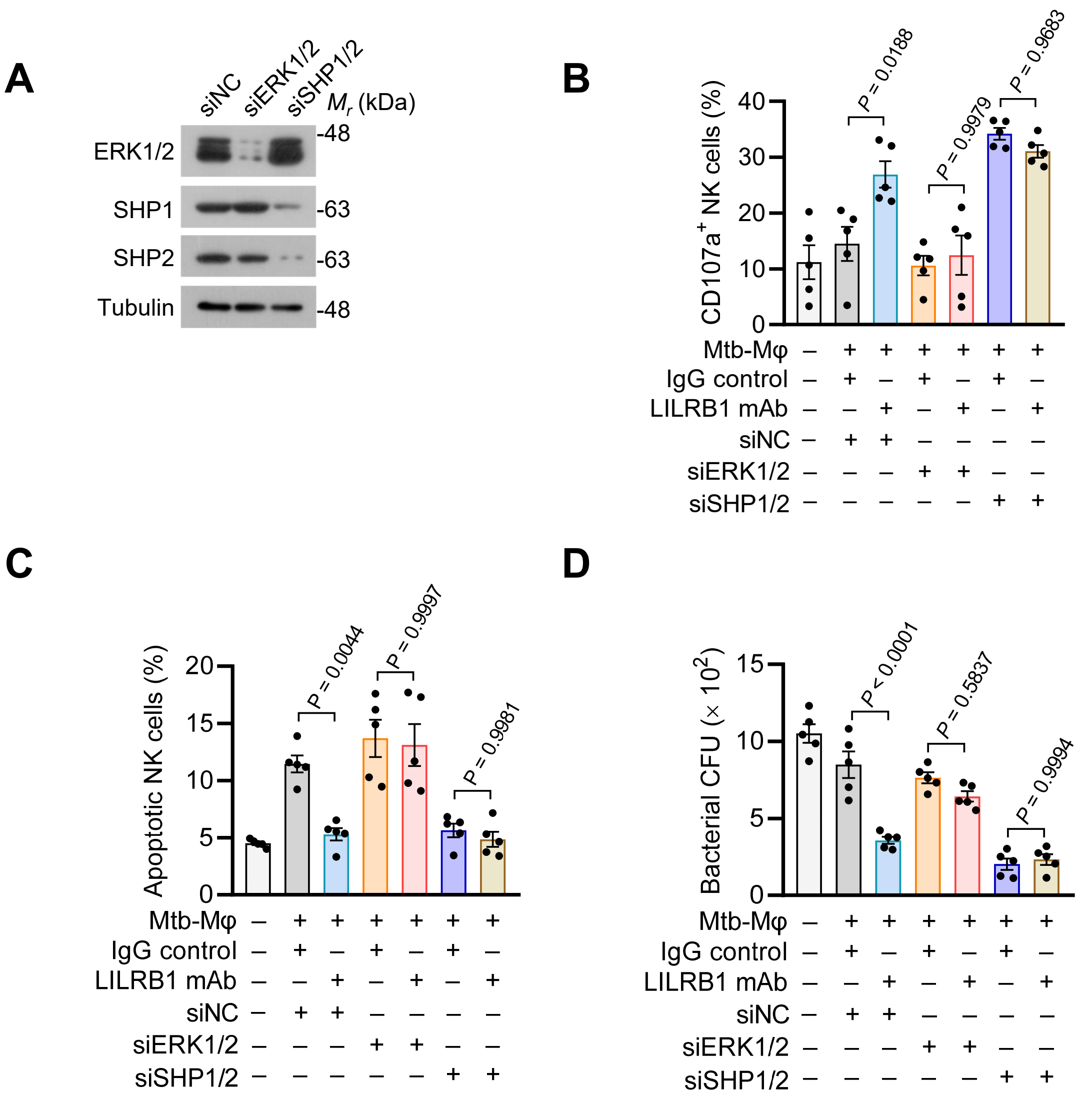

A
siNC
siERK1/2
siSHP1/2
M_r (kDa)
ERK1/2
SHP1
SHP2
Tubulin
-48
-63
-63
-48
B
CD107a$^+$ NK cells (%)
P = 0.0188
P = 0.9979
P = 0.9683
C
Apoptotic NK cells (%)
P = 0.0044
P = 0.9997
P = 0.9981
D
Bacterial CFU (× 10^2)
P < 0.0001
P = 0.5837
P = 0.9994
Mtb-Mφ – + + + + + +
IgG control – + – + – + –
LILRB1 mAb – – + – + – +
siNC – + + – – – –
siERK1/2 – – – + + – –
siSHP1/2 – – – – – + +

Appendix Figure S6. Knockdown of ERK1/2 impairs the LILRB1 blockade-enhanced anti-Mtb function of NK cells from ATB patients. (**A**) Immunoblotting for expression of ERK1/2 and SHP1/2 in ATB patient-derived NK cells. Control siRNA (siNC) or siRNA targeting *ERK1/2* (siERK1/2) or *SHP1/2* (siSHP1/2) were delivered into cells using lipid nanoparticles, and cells were lysed for analysis at 24 hours after treatment. (**B** and **C**) $CD107a^+$ (**B**) or apoptotic (**C**) cells within total NK cells. (**D**) Mtb survival in MDM-NK cell co-cultures. For (**B**–**D**), ATB patient-derived NK cells with or without knockdown of *ERK1/2* or *SHP1/2* were co-cultured with Mtb-infected MDMs (Mtb-Mφ) in the presence of anti-LILRB1 (GHI/75) mAb or IgG control for 24 hours. Data are mean ± SEM (n = 5 donors per group) in (**B**–**D**). Statistical significance was determined using one-way ANOVA with Tukey's post-hoc test. Results are representative of two independent experiments.

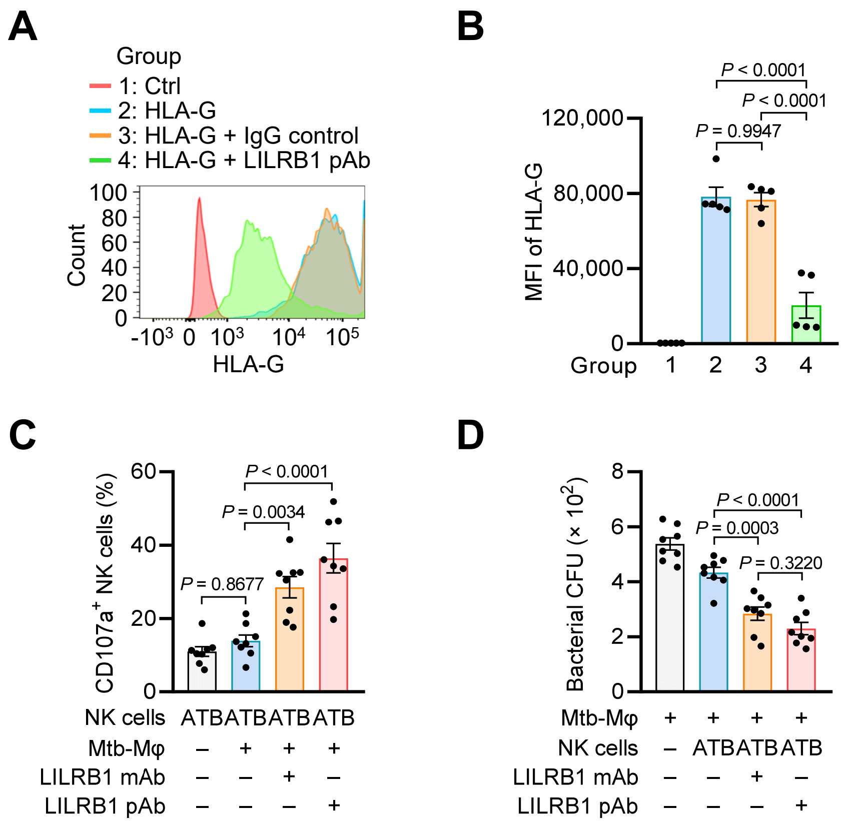
A
Group
1: Ctrl
2: HLA-G
3: HLA-G + IgG control
4: HLA-G + LILRB1 pAb
Count
HLA-G
B
MFI of HLA-G
P < 0.0001
P < 0.0001
P = 0.9947
Group 1 2 3 4
C
CD107a+ NK cells (%)
P < 0.0001
P = 0.0034
P = 0.8677
NK cells ATB ATB ATB ATB
Mtb-Mφ – + + +
LILRB1 mAb – – + –
LILRB1 pAb – – – +
D
Bacterial CFU (× 10^2)
P < 0.0001
P = 0.0003
P = 0.3220
Mtb-Mφ + + + +
NK cells – ATB ATB ATB
LILRB1 mAb – – + –
LILRB1 pAb – – – +

Appendix Figure S7. Anti-LILRB1 blocking pAb restores anti-Mtb functions of NK cells from ATB patients. (**A** and **B**) Representative histograms (**A**) and quantitation (**B**) of HLA-G binding on the LILRB1-overexpressing HEK293T cells treated with anti-LILRB1 blocking pAb or IgG control. (**C**) CD107a$^+$ cells within total NK cells from ATB patients co-cultured with Mtb-infected MDMs (Mtb-Mφ) for 24 hours with or without treatment of anti-LILRB1 (GHI/75) mAb or anti-LILRB1 blocking pAb. (**D**) Mtb survival in MDM-NK cell co-cultures after infection for 24 hours. MDMs were co-cultured with or without ATB patient-derived NK cells in the presence or absence of anti-LILRB1 (GHI/75) mAb or anti-LILRB1 blocking pAb. Data are mean ± SEM [n = 5 donors per group in (**B**) and n = 8 donors per group in (**C**, **D**)]. Statistical significance was determined using one-way ANOVA with Tukey's post-hoc test. Results are representative of two independent experiments.

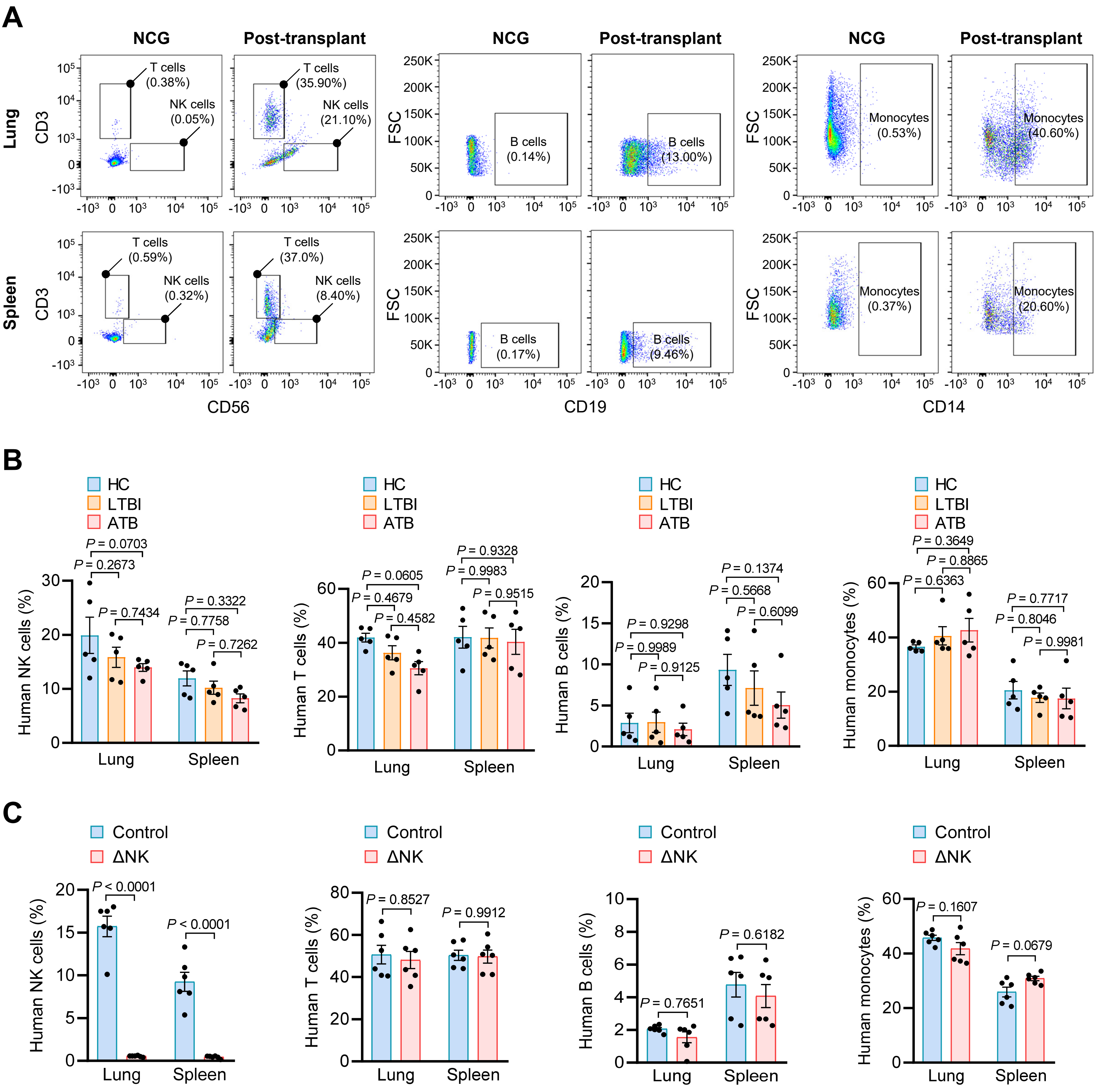
A
NCG
Post-transplant
Lung
Spleen
CD3
T cells (0.38%)
NK cells (0.05%)
T cells (35.90%)
NK cells (21.10%)
T cells (0.59%)
NK cells (0.32%)
T cells (37.0%)
NK cells (8.40%)
CD56
FSC
B cells (0.14%)
B cells (13.00%)
B cells (0.17%)
B cells (9.46%)
CD19
Monocytes (0.53%)
Monocytes (40.60%)
Monocytes (0.37%)
Monocytes (20.60%)
CD14
B
HC
LTBI
ATB
Human NK cells (%)
P = 0.0703
P = 0.2673
P = 0.7434
P = 0.3322
P = 0.7758
P = 0.7262
Human T cells (%)
P = 0.0605
P = 0.4679
P = 0.4582
P = 0.9328
P = 0.9983
P = 0.9515
Human B cells (%)
P = 0.9298
P = 0.9989
P = 0.9125
P = 0.1374
P = 0.5668
P = 0.6099
Human monocytes (%)
P = 0.3649
P = 0.8865
P = 0.6363
P = 0.7717
P = 0.8046
P = 0.9981
Lung
Spleen
C
Control
ΔNK
Human NK cells (%)
P < 0.0001
P < 0.0001
Human T cells (%)
P = 0.8527
P = 0.9912
Human B cells (%)
P = 0.7651
P = 0.6182
Human monocytes (%)
P = 0.1607
P = 0.0679
Lung
Spleen

Appendix Figure S8. Reconstitution of immuno-humanized mice deficient in NK cells. (**A**) Representative results of FACS-based analyses for each indicated human immune cell subset in NCG mice transplanted with or without PBMCs from an individual in the HC group. (**B**) Frequencies of human NK cells, T cells, B cells, and monocytes in the lungs and spleens of NCG mice transplanted with or without PBMCs from individuals in HC, LTBI, or ATB groups. (**C**) Frequencies of the indicated human immune cell subsets in NCG mice transplanted with control PBMCs or NK cell-deprived (ΔNK) PBMCs from ATB patients. The lungs and spleens of mice were collected for FACS-based analysis after transplantation for 2 weeks. Data are mean ± SEM [*n* = 5 mice per group in (**B**) and *n* = 6 mice per group in (**C**)]. Statistical significance was determined using two-way ANOVA with Tukey's post-hoc test. Results are representative of two independent experiments.

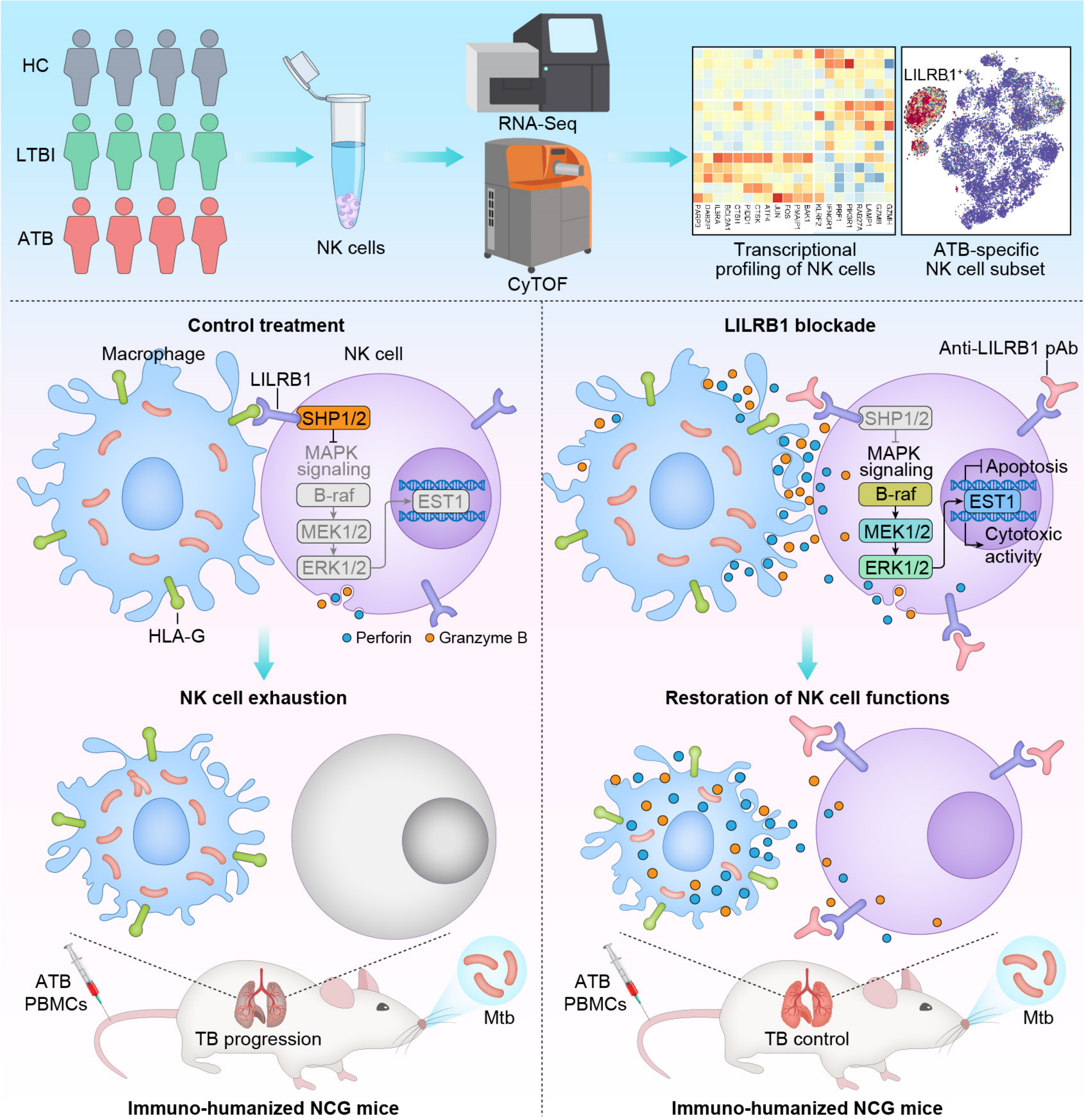
HC
LTBI
ATB
NK cells
RNA-Seq
CyTOF
Transcriptional profiling of NK cells
LILRB1⁺
ATB-specific NK cell subset
Control treatment
Macrophage
NK cell
LILRB1
SHP1/2
MAPK signaling
B-raf
MEK1/2
ERK1/2
EST1
HLA-G
Perforin
Granzyme B
NK cell exhaustion
LILRB1 blockade
Anti-LILRB1 pAb
SHP1/2
MAPK signaling
B-raf
MEK1/2
ERK1/2
EST1
Apoptosis
Cytotoxic activity
Restoration of NK cell functions
ATB PBMCs
TB progression
Mtb
Immuno-humanized NCG mice
ATB PBMCs
TB control
Mtb
Immuno-humanized NCG mice

Appendix Figure S9. Schematic model of this study. The model shows the mechanism by which LILRB1 blockade restores functions of exhausted NK cells from ATB patients to enhance host anti-Mtb protective immune responses.

Appendix Table S1. Antibodies used for mass cytometry analysis in this study.

Label	Marker	Clone	Manufacturer	Dilution
89Y	CD45	HI30	BioLegend	1:200
115ln	CD3	UCHTI	BioLegend	1:200
139La	IFN-γ	B27	BioLegend	1:100
141Pr	CD56	NCAM16.2	BD Biosciences	1:800
142Nd	CD19	HIB19	BioLegend	1:400
143Nd	CD27	O323	BioLegend	1:400
144Nd	CD14	M5E2	BioLegend	1:50
145Nd	KIR2DL2	GL183	Beckman	1:50
146Nd	CD226	DX11	BD Biosciences	1:25
147Sm	LILRB1	GHI/75	Biolegend	1:25
148Nd	CD69	FN50	Biolegend	1:100
149Sm	KIR3DL1	DX9	BD Biosciences	1:800
151Eu	KIR2DS4	179315	R&D Systems	1:25
152Sm	TNF-α	Mab11	BioLegend	1:100
153Eu	KLRB1	HP-3G10	BioLegend	1:50
154Sm	TIGIT	A15153G	BioLegend	1:25
155Gd	NKp46	9E2	BioLegend	1:50
156Gd	NKG2D	1D11	BioLegend	1:25
157Gd	NKG2C	S19005E	BioLegend	1:100
158Gd	2B4	2-69	BioLegend	1:25
159Tb	CCL4	24006	R&D Systems	1:100
160Gd	CXCR6	56811	R&D Systems	1:100
161Dy	KIR2DL4	mAb#33	Invitrogen	1:50
162Dy	KIR2DL5	UP-R1	Invitrogen	1:50
163Dy	NKG2A	131411	R&D Systems	1:25
164Dy	KIR2DL3	180701	R&D Systems	1:25
165Ho	CD66b	6/40c	BioLegend	1:800
166Er	PRF1	B-D48	Abcam	1:20
167Er	CD94	HP-3D9	BD Biosciences	1:200
168Er	GM-CSF	BVD2-21C11	BioLegend	1:100
169Tm	CD122	TU27	BioLegend	1:25
170Er	CD127	A019D5	BioLegend	1:100
171Yb	NKp30	P30-15	BioLegend	1:25
172Yb	NKp44	P44-8	BioLegend	1:50
173Yb	GZMB	QA16A02	BioLegend	1:100
174Yb	PD-1	EH12.2H7	BioLegend	1:100
175Lu	CD16	3G8	BioLegend	1:200
176Yb	HLA-DR	L243	BioLegend	1:200
197Au	CD4	RPA-T4	BioLegend	1:400
198Pt	CD8	RPA-T8	BioLegend	1:800
209Bi	ITGAL	HI111	BioLegend	1:800
