# Supplementary figures and images for: LILRB1-HLA-G axis defines a checkpoint driving natural killer cell exhaustion in tuberculosis
Source: EMBO Mol Med. 2024 Jul 19;16(8):1. doi: 10.1038/s44321-024-00106-1 (PMC11319715; doi:10.1038/s44321-024-00106-1)

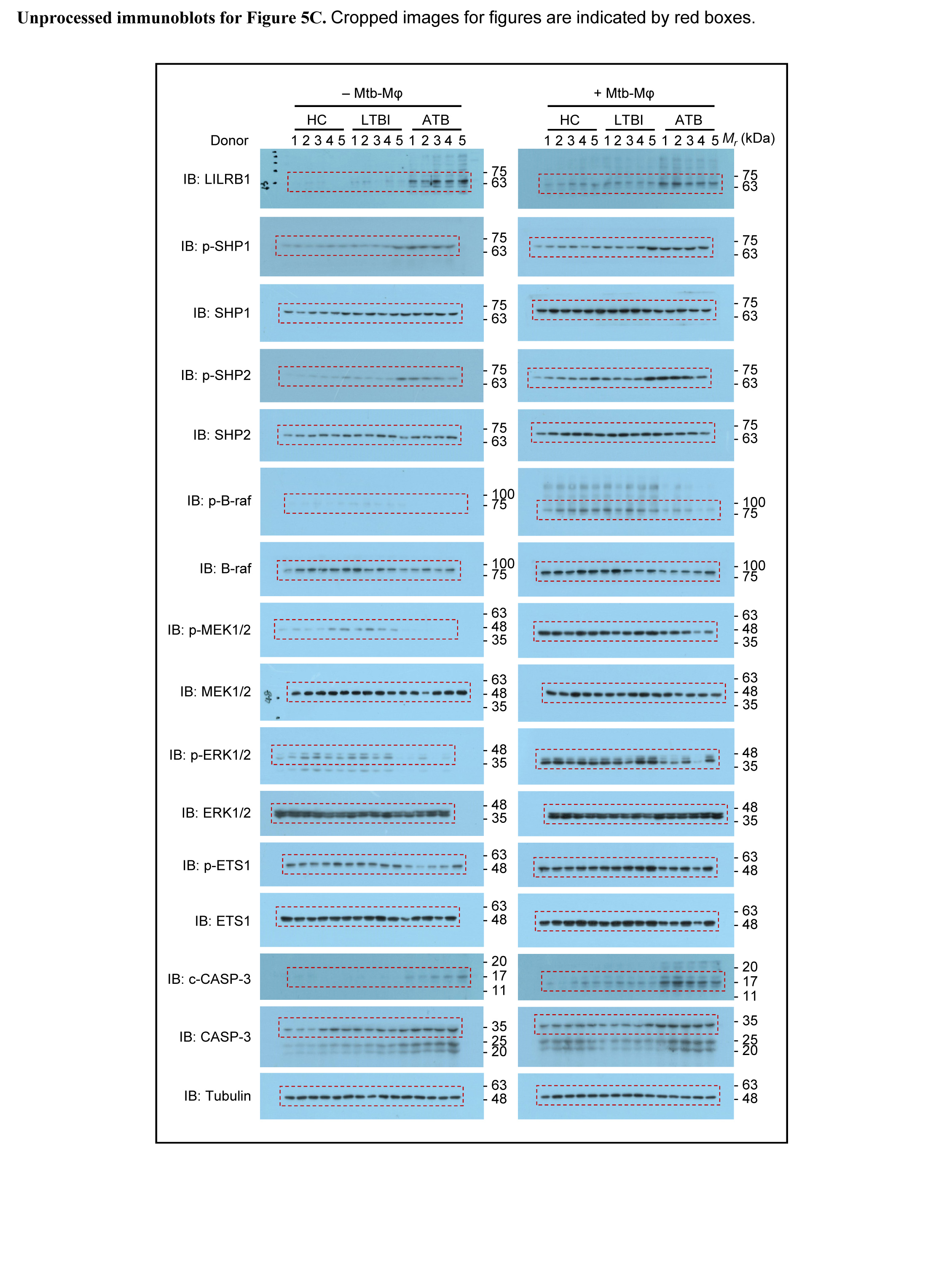

Supplement: Supplementary file 10 — Source data Fig. 5 [file 44321_2024_106_MOESM10_ESM.zip › Figure 5/Figure 5C/5C Uncropped images for blots.jpg]

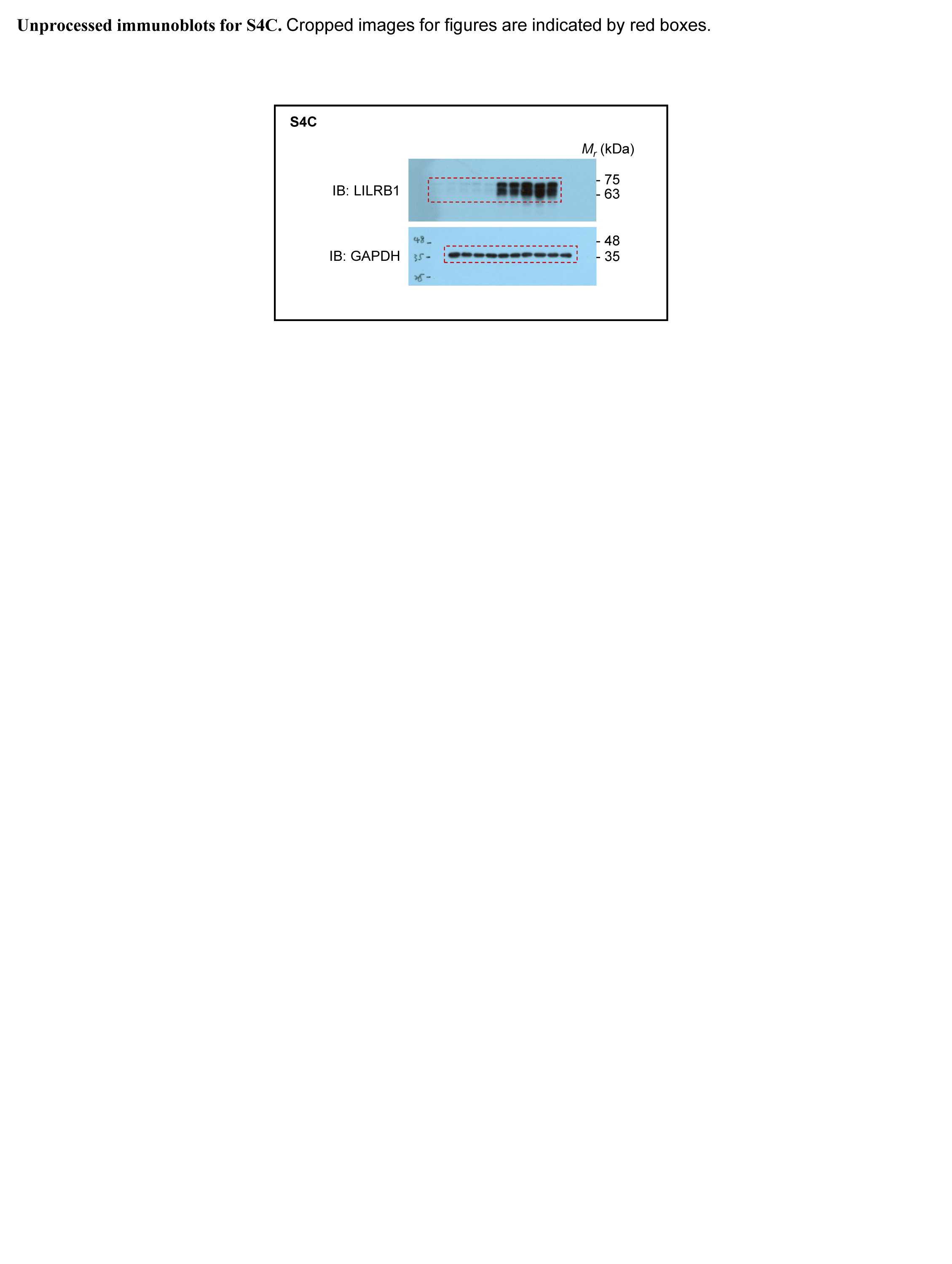

Supplement: Supplementary file 14 — Source Data for EV and Appendix figures [file 44321_2024_106_MOESM14_ESM.zip › Source data for EV and Appendix figures/Appendix Figure S4/Appendix Figure S4C/S4C Uncropped images for blots.jpg]

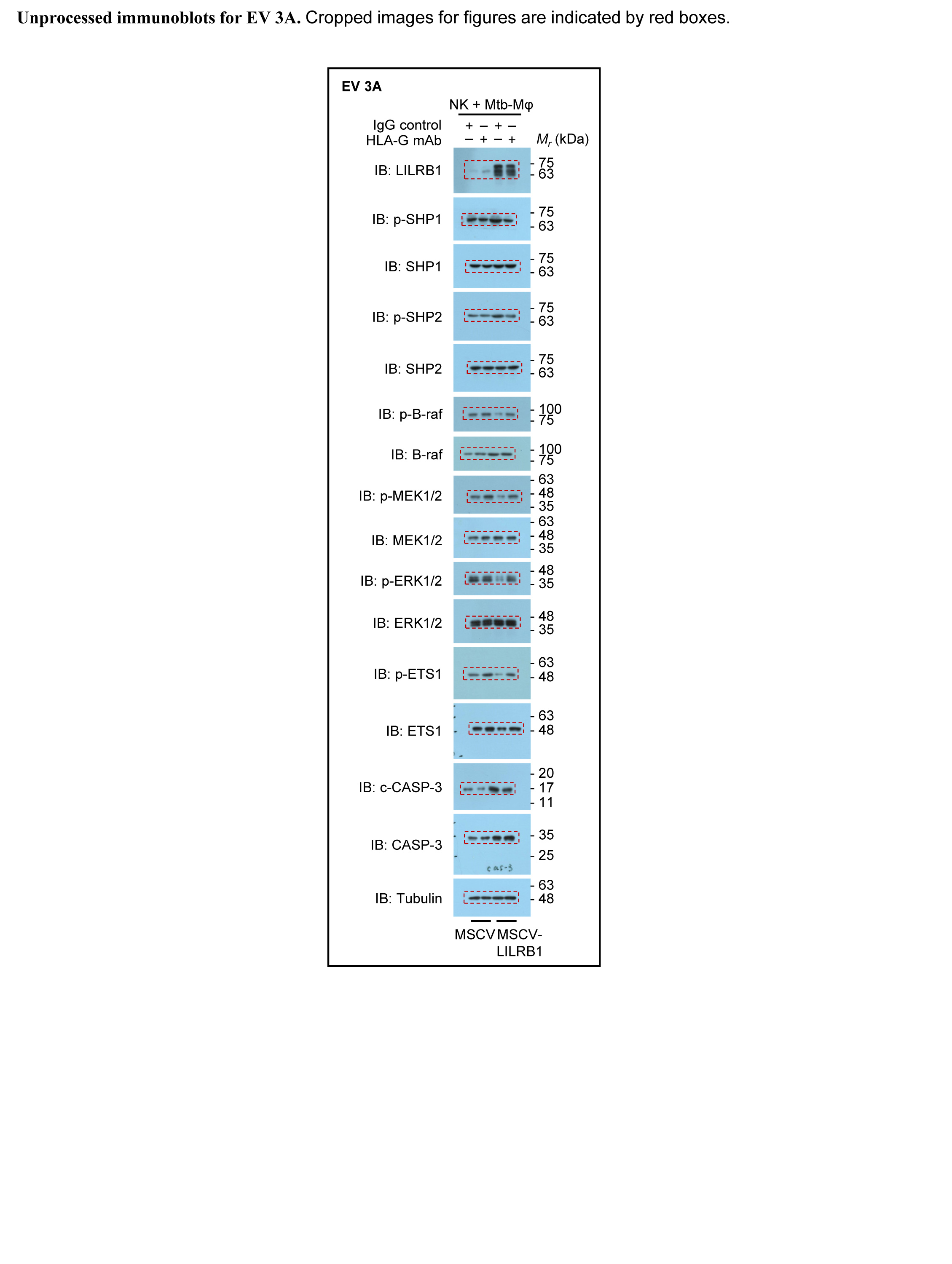

Supplement: Supplementary file 14 — Source Data for EV and Appendix figures [file 44321_2024_106_MOESM14_ESM.zip › Source data for EV and Appendix figures/Expanded View Figure 3/Expanded View Figure 3A/EV3A Uncropped images for blots.jpg]
