# Supplementary material for: LILRB1-HLA-G axis defines a checkpoint driving natural killer cell exhaustion in tuberculosis
Source: EMBO Mol Med. 2024 Jul 19;16(8):1. doi: 10.1038/s44321-024-00106-1 (PMC11319715; doi:10.1038/s44321-024-00106-1)
Supplement: Supplementary file 15 — Expanded View Figures [file 44321_2024_106_MOESM15_ESM.pdf]

Expanded View Figures

Figure EV1. Frequencies of immune cell subsets in individuals from HC, LTBI, and ATB groups.

(**A**) Flow diagram summarizing the participant recruitment in this study. See “Methods” and Dataset EV1 for details of inclusion criteria and demographic information of participants included, respectively. (**B**) Representative results of fluorescence-activated cell sorting (FACS)-based analyses for frequencies of immune cell subsets in peripheral blood of individuals from HC, LTBI, and ATB groups. Each immune cell subsets were characterized using standard cell subset definitions. NK cell gating: FSC, SSC, $CD3^-$, $CD19^-$, $CD56^{bright/dim}$; T cell gating: FSC, SSC, $CD3^+$, $CD19^-$, $CD56^-$; B cell gating: FSC, SSC, $CD3^-$, $CD19^+$, $CD56^-$; monocyte gating: FSC, SSC, $CD3^-$, $CD19^-$, $CD14^+$. (**C**) The number of immune cell subsets in peripheral blood of individuals (n) from HC, LTBI, and ATB groups. Statistical significance was determined using one-way ANOVA with Tukey’s post-hoc test (**C**). Source data are available online for this figure.

© The Author(s)

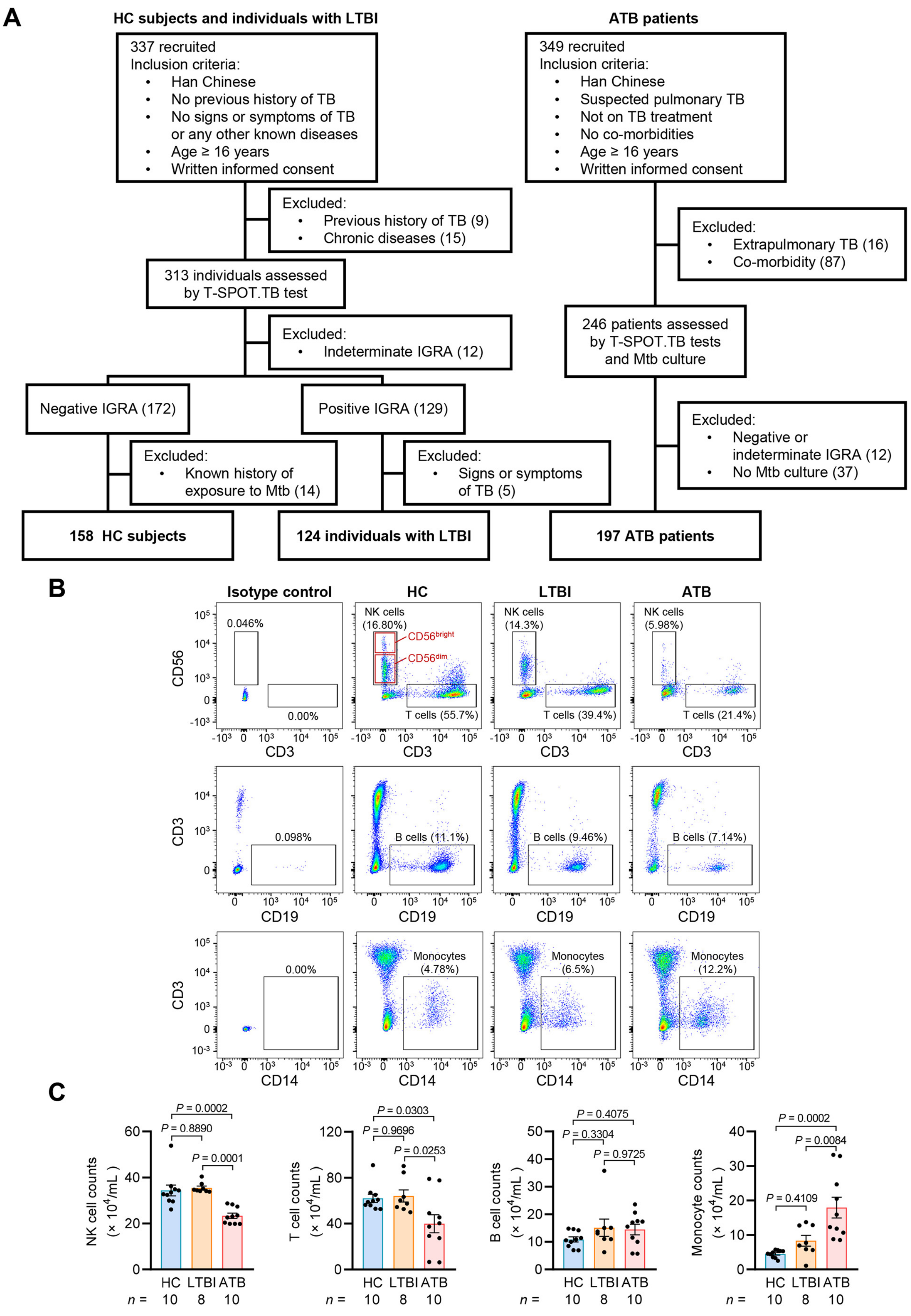
A
HC subjects and individuals with LTBI
337 recruited
Inclusion criteria:
Han Chinese
No previous history of TB
No signs or symptoms of TB or any other known diseases
Age ≥ 16 years
Written informed consent
Excluded:
Previous history of TB (9)
Chronic diseases (15)
313 individuals assessed by T-SPOT.TB test
Excluded:
Indeterminate IGRA (12)
Negative IGRA (172)
Positive IGRA (129)
Excluded:
Known history of exposure to Mtb (14)
Excluded:
Signs or symptoms of TB (5)
158 HC subjects
124 individuals with LTBI
ATB patients
349 recruited
Inclusion criteria:
Han Chinese
Suspected pulmonary TB
Not on TB treatment
No co-morbidities
Age ≥ 16 years
Written informed consent
Excluded:
Extrapulmonary TB (16)
Co-morbidity (87)
246 patients assessed by T-SPOT.TB tests and Mtb culture
Excluded:
Negative or indeterminate IGRA (12)
No Mtb culture (37)
197 ATB patients
B
Isotype control
HC
LTBI
ATB
CD56
CD3
0.046%
0.00%
NK cells (16.80%)
CD56bright
CD56dim
T cells (55.7%)
NK cells (14.3%)
T cells (39.4%)
NK cells (5.98%)
T cells (21.4%)
CD19
0.098%
B cells (11.1%)
B cells (9.46%)
B cells (7.14%)
CD14
0.00%
Monocytes (4.78%)
Monocytes (6.5%)
Monocytes (12.2%)
C
NK cell counts (× 10^4/mL)
P = 0.0002
P = 0.8890
P = 0.0001
T cell counts (× 10^4/mL)
P = 0.0303
P = 0.9696
P = 0.0253
B cell counts (× 10^4/mL)
P = 0.4075
P = 0.3304
P = 0.9725
Monocyte counts (× 10^4/mL)
P = 0.0002
P = 0.0084
P = 0.4109
HC LTBI ATB
n = 10 8 10

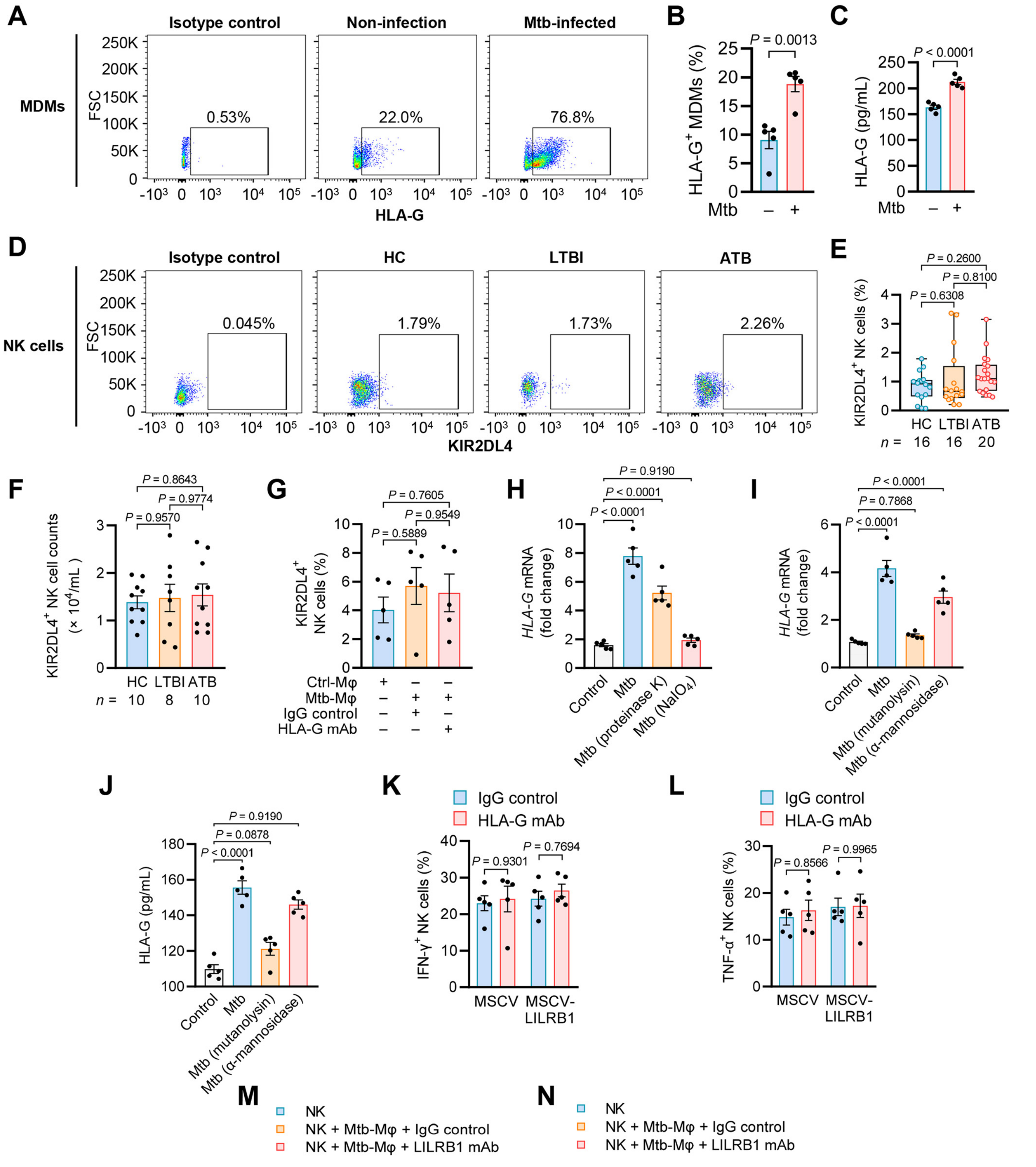
A
Isotype control
Non-infection
Mtb-infected
MDMs
FSC
HLA-G
0.53%
22.0%
76.8%
B
HLA-G+ MDMs (%)
P = 0.0013
Mtb
C
HLA-G (pg/mL)
P < 0.0001
Mtb
D
Isotype control
HC
LTBI
ATB
NK cells
FSC
KIR2DL4
0.045%
1.79%
1.73%
2.26%
E
KIR2DL4+ NK cells (%)
P = 0.2600
P = 0.8100
P = 0.6308
HC LTBI ATB
n = 16 16 20
F
KIR2DL4+ NK cell counts (× 10^4/mL)
P = 0.8643
P = 0.9774
P = 0.9570
HC LTBI ATB
n = 10 8 10
G
KIR2DL4+ NK cells (%)
P = 0.7605
P = 0.9549
P = 0.5889
Ctrl-Mφ
Mtb-Mφ
IgG control
HLA-G mAb
H
HLA-G mRNA (fold change)
P = 0.9190
P < 0.0001
P < 0.0001
Control
Mtb
Mtb (proteinase K)
Mtb (NaIO4)
I
HLA-G mRNA (fold change)
P < 0.0001
P = 0.7868
P < 0.0001
Control
Mtb
Mtb (mutanolysin)
Mtb (α-mannosidase)
J
HLA-G (pg/mL)
P = 0.9190
P = 0.0878
P < 0.0001
Control
Mtb
Mtb (mutanolysin)
Mtb (α-mannosidase)
K
IgG control
HLA-G mAb
IFN-γ+ NK cells (%)
P = 0.9301
P = 0.7694
MSCV
MSCV-LILRB1
L
IgG control
HLA-G mAb
TNF-α+ NK cells (%)
P = 0.8566
P = 0.9965
MSCV
MSCV-LILRB1
M
NK
NK + Mtb-Mφ + IgG control
NK + Mtb-Mφ + LILRB1 mAb
IFN-γ+ NK cells (%)
P = 0.8853
P = 0.9587
P = 0.9898
HC LTBI ATB
N
NK
NK + Mtb-Mφ + IgG control
NK + Mtb-Mφ + LILRB1 mAb
TNF-α+ NK cells (%)
P = 0.9816
P = 0.9831
P = 0.9711
HC LTBI ATB

◀ **Figure EV2. Mtb infection increases HLA-G production in macrophages.**

(**A**) Representative results of FACS-based analysis for percentages of HLA-G^{+} MDMs from HC group after infection with or without Mtb for 3 days. (**B**, **C**) Percentages of HLA-G^{+} cells (**B**) and supernatant levels of soluble HLA-G (**C**) in MDMs treated as in (**A**). (**D**) Representative results of FACS-based analysis for percentages of KIR2DL4^{+} cells within total NK cells from the peripheral blood of individuals in HC, LTBI, or ATB groups. (**E**) Percentages of KIR2DL4^{+} cells within total NK cells in the peripheral blood of individuals (n) from HC, LTBI, or ATB groups. Box-whisker plot indicates the interquartile range (box), the median value (line within the box), and the maximum and minimum value (whiskers). (**F**) The number of KIR2DL4^{+} NK cells in the peripheral blood of individuals (n) from HC, LTBI, or ATB groups. (**G**) Percentages of KIR2DL4^{+} cells within NK cells. NK cells were co-cultured with Mtb-Mφ or control MDMs (Ctrl-Mφ) for 3 days with treatment of anti-HLA-G (87G) mAb or IgG control. (**H**, **I**) Quantitative PCR (qPCR) analysis of *HLA-G* mRNA in MDMs. MDMs were incubated with Mtb at a MOI of 5 for 3 days. Mtb was pretreated with or without proteinase K (to remove Mtb surface proteins), $NaIO_4$ (to remove Mtb surface carbohydrate residues), mutanolysin (to remove peptidoglycan), or α-mannosidase (to remove lipomannan and lipoarabinomannan). (**J**) Enzyme-linked immunosorbent assay (ELISA) of supernatant HLA-G produced by MDMs treated as in (**I**). (**K**, **L**) Percentages of IFN-γ^{+} (**K**) or TNF-α^{+} (**L**) cells within total NK cells. HC donor-derived NK cells with overexpression of MSCV or MSCV-LILRB1 were co-cultured with Mtb-Mφ for 24 h in the presence of anti-HLA-G (87G) antibody or IgG control. (**M**, **N**) Percentages of IFN-γ^{+} (**M**) or TNF-α^{+} (**N**) cells in MDM-NK cell co-cultures. NK cells from each indicated group were co-cultured with Mtb-Mφ for 24 h in the presence of anti-LILRB1 (GHI/75) mAb or IgG control. Data are mean ± SEM ($n = 5$ donors per group) in (**G–N**). Statistical significance was determined using two-tailed t-test (**B**, **C**), one-way ANOVA (**E–J**), and two-way ANOVA (**K–N**) with Tukey's post-hoc test. Results are representative of three independent experiments. Source data are available online for this figure.

 © The Author(s)

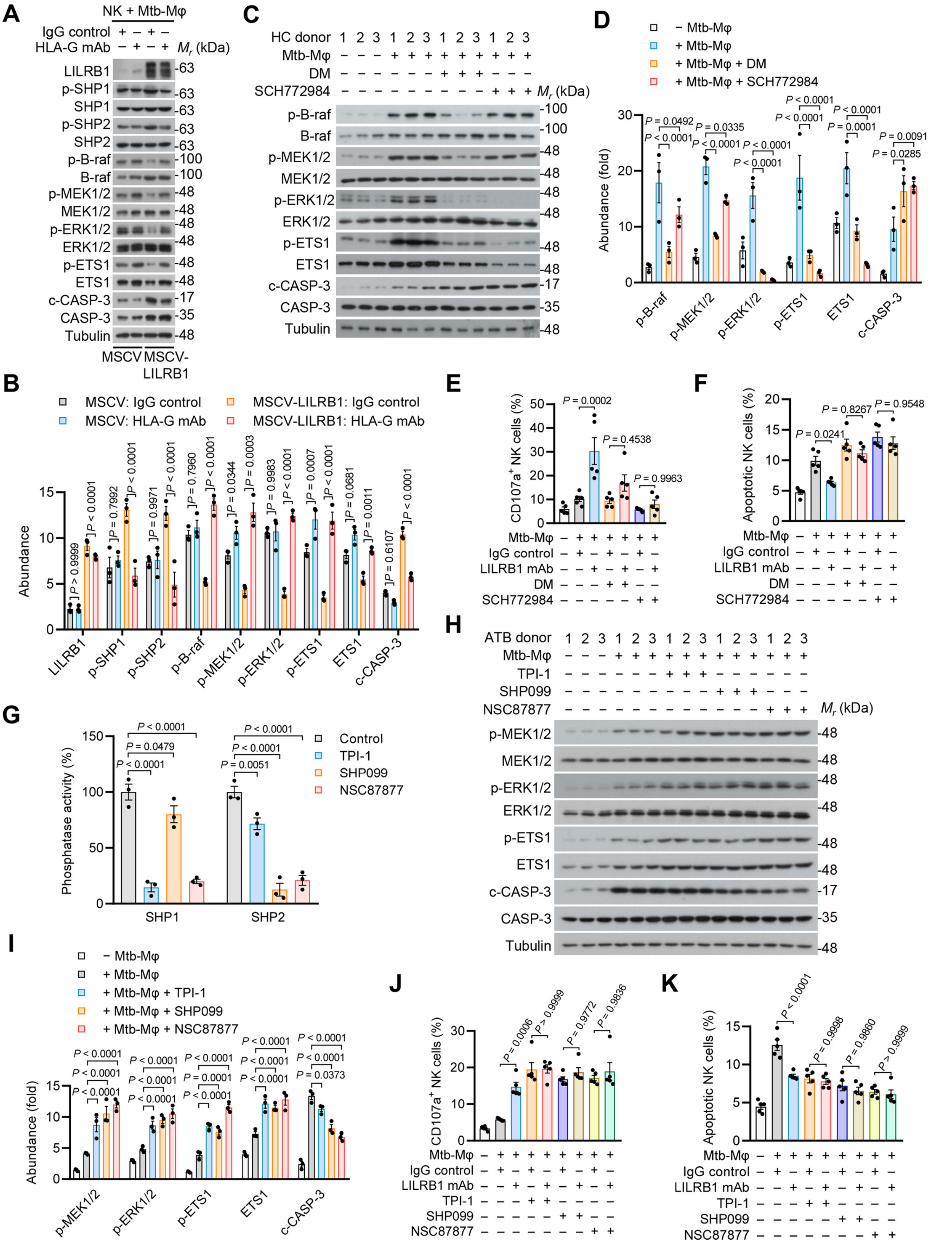
A
B
C
D
E
F
G
H
I
J
K
NK + Mtb-Mφ
IgG control
HLA-G mAb
MSCV
MSCV-LILRB1
HC donor
ATB donor
Mtb-Mφ
DM
SCH772984
TPI-1
SHP099
NSC87877
LILRB1 mAb
Abundance
Abundance (fold)
CD107a+ NK cells (%)
Apoptotic NK cells (%)
Phosphatase activity (%)
Control
SHP1
SHP2
LILRB1
p-SHP1
p-SHP2
p-B-raf
B-raf
p-MEK1/2
MEK1/2
p-ERK1/2
ERK1/2
p-ETS1
ETS1
c-CASP-3
CASP-3
Tubulin

Figure EV3. Inhibition of SHP1/2 increases the activation of MEK1/2-ERK1/2-ETS1 signaling axis in NK cells.

(**A**, **B**) Immunoblotting (**A**) and quantitation (**B**) for expression of indicated proteins in HC-donor-derived NK cells with overexpression of MSCV or MSCV-LILRB1. Cells were co-cultured with Mtb-infected MDMs for 24 h in the presence of anti-HLA-G (87G) antibody or IgG control. (**C**, **D**) Immunoblotting (**C**) and quantitation (**D**) for expression of indicated proteins in HC-donor-derived NK cells co-cultured with or without Mtb-infected MDMs (Mtb-Mφ) and treated with or without dabrafenib mesylate (DM) or SCH772984 for 24 h. (**E**, **F**) Percentages of $CD107a^{+}$ (**E**) and apoptotic (**F**) cells within total NK cells from ATB patients. NK cells were co-cultured with Mtb-infected MDMs and treated with the indicated inhibitors in the presence of anti-LILRB1 (GHI/75) mAb or IgG control for 24 h. (**G**) Phosphatase activity of SHP1 and SHP2 in NK cells derived from ATB patients. Cells were treated with or without 0.5 μM TPI-1, SHP099, or NSC87877 for 24 h. (**H**, **I**) Immunoblotting (**H**) and quantitation (**I**) for expression of indicated proteins in ATB patient-derived NK cells. Cells were co-cultured with or without Mtb-Mφ in the presence or absence of indicated inhibitors (0.5 μM each) for 24 h. (**J**, **K**) Percentages of $CD107a^{+}$ (**J**) and apoptotic (**K**) cells within total NK cells from ATB patients. NK cells were co-cultured with Mtb-infected MDMs and treated with the indicated inhibitors in the presence of anti-LILRB1 (GHI/75) mAb or IgG control for 24 h. Data are mean ± SEM [$n = 3$ donors per group in (**B**, **D**, **G**, **I**) and $n = 5$ donors per group in (**E**, **F**, **J**, **K**)]. Statistical significance was determined using two-way ANOVA (**B**, **D**, **G**, **I**) and one-way ANOVA (**E**, **F**, **J**, **K**) with Tukey's post-hoc test. Results are representative of three independent experiments. Source data are available online for this figure.

 © The Author(s)

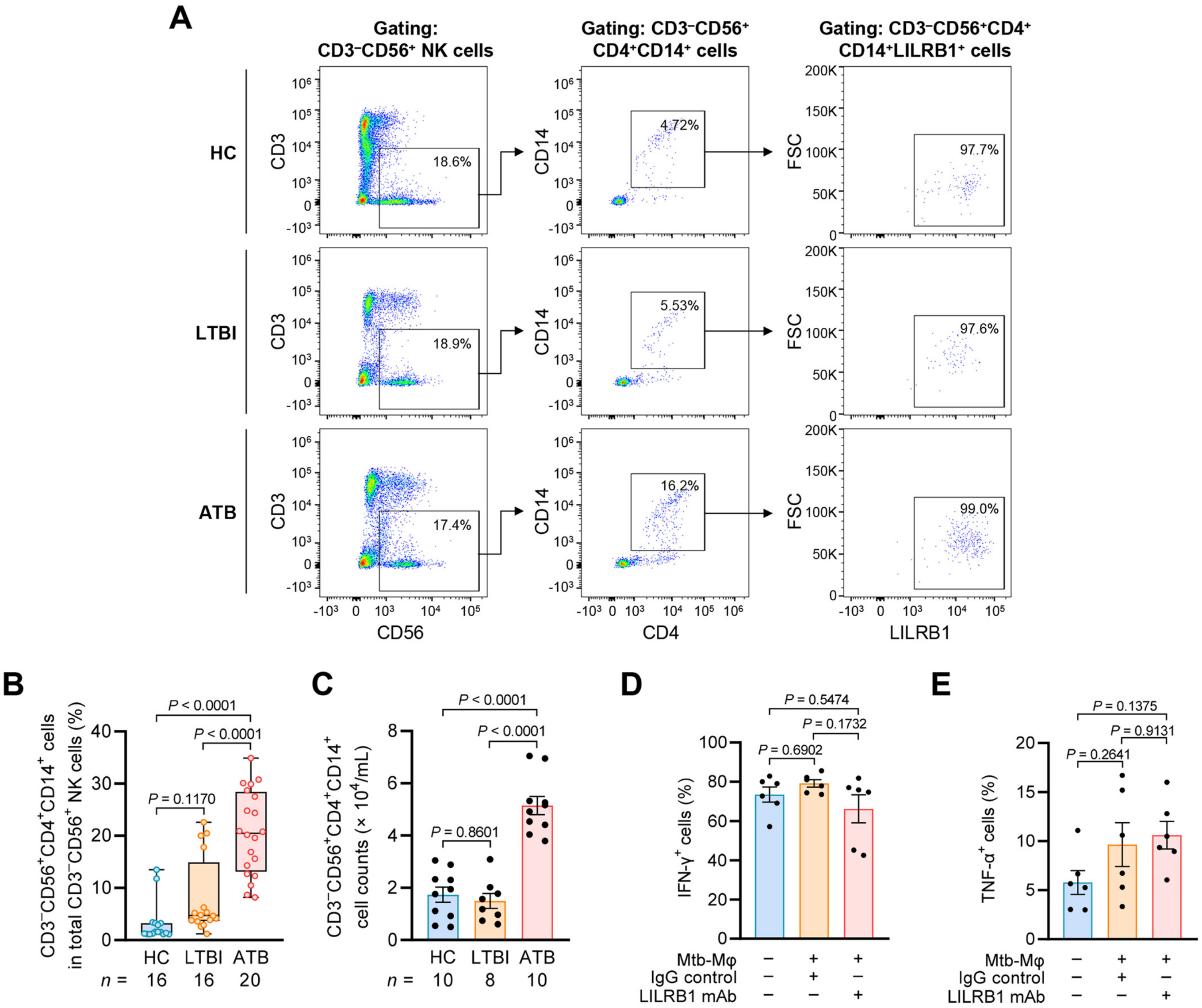

Figure EV4. The frequency of CD3⁻CD56⁺CD4⁺CD14⁺ cell subset is increased in TB patients.

(**A**) Representative results of FACS-based analysis for percentages of CD3⁻CD56⁺CD4⁺CD14⁺ cells within total CD3⁻CD56⁺ NK cells from the peripheral blood of individuals in HC, LTBI, or ATB groups. (**B**) Percentages of CD3⁻CD56⁺CD4⁺CD14⁺ cells within total CD3⁻CD56⁺ NK cells in the peripheral blood of individuals (*n*) from HC, LTBI, or ATB groups. Box-whisker plot indicates the interquartile range (box), the median value (line within the box), and the maximum and minimum value (whiskers). (**C**) The number of CD3⁻CD56⁺CD4⁺CD14⁺ cells in the peripheral blood of individuals (*n*) from HC, LTBI, or ATB groups. (**D**, **E**) Percentages of IFN-γ⁺ (**D**) and TNF-α⁺ (**E**) cells in total CD3⁻CD56⁺CD4⁺CD14⁺ cells. CD3⁻CD56⁺CD4⁺CD14⁺ cells were co-cultured with or without Mtb-Mφ at a ratio of 3:1 for 24 h in the presence of anti-LILRB1 (GHI/75) mAb or IgG control. Data are mean ± SEM ($n = 6$ donors per group) in (**D**, **E**). Statistical significance was determined using one-way ANOVA with Tukey's post-hoc test for (**B**–**E**). Results are representative of three independent experiments. Source data are available online for this figure.

© The Author(s)

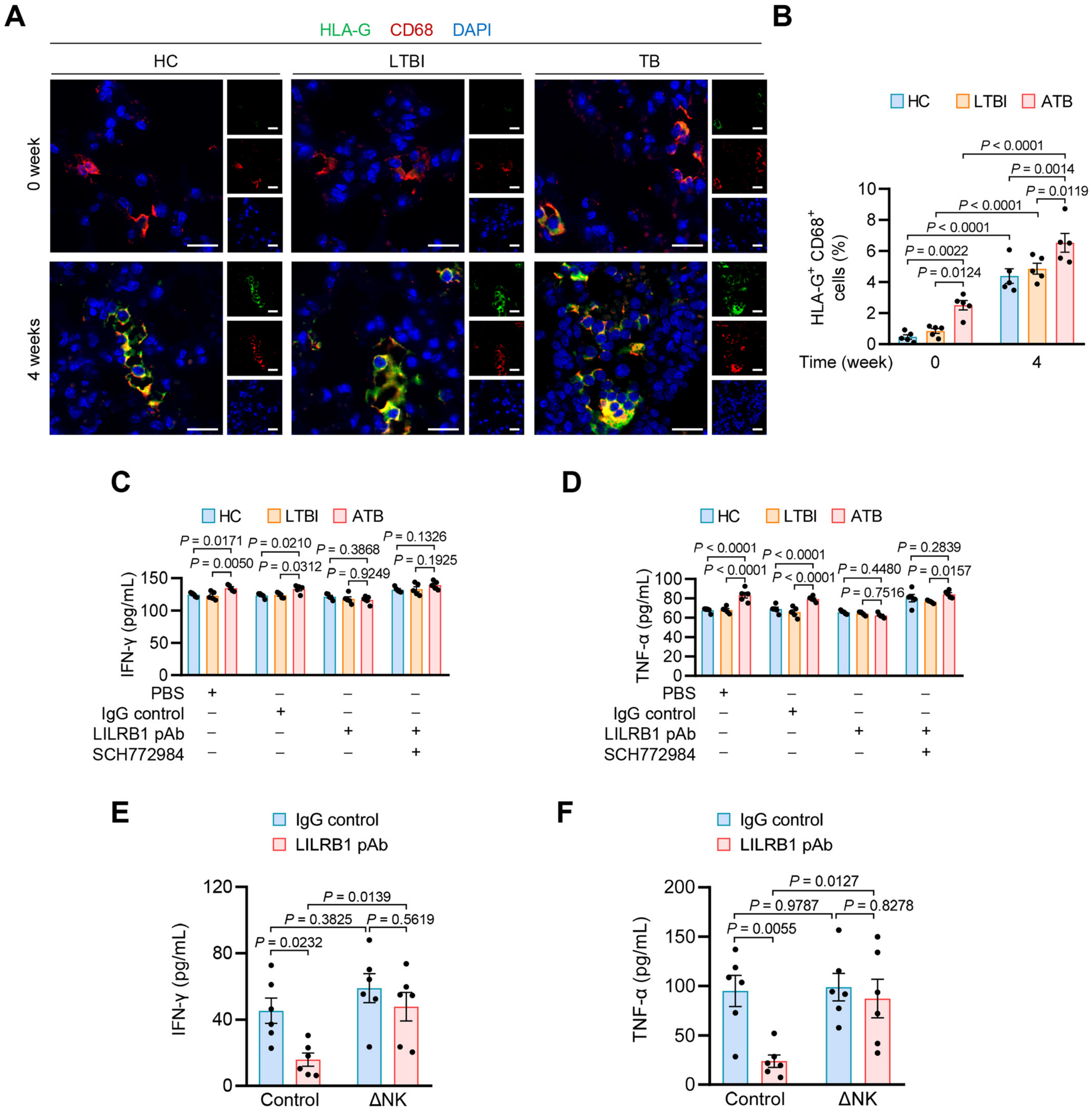

Figure EV5. Mtb infection increases macrophage expression of HLA-G in the lung of immuno-humanized mice.

(**A**) Representative images for cells stained with antibodies against HLA-G (green) and CD68 (red) in the lungs of NCG mice after infection with Mtb for 0–4 weeks. Nuclei were stained with DAPI (blue). Scale bars, 20 μm. (**B**) Quantitation of HLA-G expression in $CD68^+$ cells. (**C–F**) Quantitation of IFN-γ (**C**, **E**) and TNF-α (**D**, **F**) in the lungs of NCG mice. For (**A–D**), mice ($n = 5$ per group) were transplanted with PBMCs from individuals in HC, LTBI, or ATB groups. Two weeks later, mice were infected with Mtb by aerosol (~100 CFUs) for 4 weeks with treatment of PBS (as control), IgG control, or anti-LILRB1 blocking pAb every 3 days, together with the treatment of corn oil (as control) or SCH772984 every 2 days (see Fig. 7A). For (**E**, **F**), control PBMCs or NK cell-depleted (ΔNK) PBMCs from ATB patients were transplanted to NCG mice ($n = 6$ per group). Two weeks later, mice were infected with Mtb by aerosol (~100 CFUs) for 4 weeks with treatment of IgG control or anti-LILRB1 blocking pAb every 3 days (see Fig. 8A). Data are mean ± SEM [$n = 5$ mice per group in (**B–D**) and $n = 6$ mice per group in (**E**, **F**)]. Statistical significance was determined using two-way ANOVA with Tukey's post-hoc test for (**B–F**). Results are representative of two independent experiments. Source data are available online for this figure.

 © The Author(s)
